# Comprehensive analysis and immune landscape of chemokines- and chemokine receptors-based signature in hepatocellular carcinoma

**DOI:** 10.3389/fimmu.2023.1164669

**Published:** 2023-07-20

**Authors:** Ze Zhang, Mingsong Mao, Fangzhou Wang, Yao Zhang, Jihang Shi, Lei Chang, Xiaolin Wu, Zhenpeng Zhang, Ping Xu, Shichun Lu

**Affiliations:** ^1^ Medical School of Chinese People’s Liberation Army (PLA), Beijing, China; ^2^ Faculty of Hepato-Pancreato-Biliary Surgery, Chinese PLA General Hospital, Beijing, China; ^3^ Institute of Hepatobiliary Surgery of Chinese PLA, Beijing, China; ^4^ Key Laboratory of Digital Hepatobiliary Surgery, PLA, Beijing, China; ^5^ School of Basic Medical Sciences, Anhui Medical University, Hefei, China; ^6^ State Key Laboratory of Proteomics, National Center for Protein Sciences (Beijing), Research Unit of Proteomics and Research and Development of New Drug of Chinese Academy of Medical Sciences, Beijing Proteome Research Center, Institute of Lifeomics, Beijing, China; ^7^ School of Medicine, Guizhou University, Guiyang, Guizhou, China

**Keywords:** hepatocellular carcinoma, chemokine, tumor immune microenvironment, prognosis, single-cell RNA-seq

## Abstract

**Background:**

Despite encouraging results from immunotherapy combined with targeted therapy for hepatocellular carcinoma (HCC), the prognosis remains poor. Chemokines and their receptors are an essential component in the development of HCC, but their significance in HCC have not yet been fully elucidated. We aimed to establish chemokine-related prognostic signature and investigate the association between the genes and tumor immune microenvironment (TIME).

**Methods:**

342 HCC patients have screened from the TCGA cohort. A prognostic signature was developed using least absolute shrinkage and selection operator regression and Cox proportional risk regression analysis. External validation was performed using the LIHC-JP cohort deployed from the ICGC database. Single-cell RNA sequencing (scRNA-seq) data from the GEO database. Two nomograms were developed to estimate the outcome of HCC patients. RT-qPCR was used to validate the differences in the expression of genes contained in the signature.

**Results:**

The prognostic signature containing two chemokines-(CCL14, CCL20) and one chemokine receptor-(CCR3) was successfully established. The HCC patients were stratified into high- and low-risk groups according to their median risk scores. We found that patients in the low-risk group had better outcomes than those in the high-risk group. The results of univariate and multivariate Cox regression analyses suggested that this prognostic signature could be considered an independent risk factor for the outcome of HCC patients. We discovered significant differences in the infiltration of various immune cell subtypes, tumor mutation burden, biological pathways, the expression of immune activation or suppression genes, and the sensitivity of different groups to chemotherapy agents and small molecule-targeted drugs in the high- and low-risk groups. Subsequently, single-cell analysis results showed that the higher expression of CCL20 was associated with HCC metastasis. The RT-qPCR results demonstrated remarkable discrepancies in the expression of CCL14, CCL20, and CCR3 between HCC and its paired adjacent non-tumor tissues.

**Conclusion:**

In this study, a novel prognostic biomarker explored in depth the association between the prognostic model and TIME was developed and verified. These results may be applied in the future to improve the efficacy of immunotherapy or targeted therapy for HCC.

## Introduction

Hepatocellular carcinoma (HCC) is the third leading contributor to mortality from cancer globally, with a five-year overall survival rate of about 18% and 830,000 deaths from the disease each year (1). Despite substantial advances in local and systemic therapy for intermediate to advanced HCC, in particular, immunotherapy and molecular targeted therapy, most patients may not respond or develop resistance to the drugs and eventually died of the disease (2). Therefore, there is still an emergency to explore more effective systemic therapies, as well as predictable biomarkers, to enable personalized and cost-effective treatment stratification.

Chemokines are a class of 8- to 12-kDa secreted proteins that can be classified into four categories XC, CC, CXC, and CX3C (3). Their main functions include the regulation of target cell migration (chemotaxis), adhesive properties, cell development, cellular localization, and cell-cell interfaces which serve an instrumental part in intracellular homeostasis, and pathological processes, in particular tumorigenesis (4). Chemokines contribute to tumor immunity in a variety of aspects, as they are involved in the localization and migration patterns of immune cells in the lymphoid tissue and tumor immune microenvironment (TIME) and directly shape the immune response (5). In general, tumor-associated mesenchymal and cancer cells could unleash a diverse array of chemokines, resulting in the recruitment and the activation of various cell types, which in turn mediate the equilibrium between anti-tumor and pro-tumor reactions (6). Several chemokine receptor antagonists have shown antitumor effectiveness in preclinical studies in various cancers, including hepatocellular carcinoma (7, 8). Recent studies have also shown that the combination of chemokine antagonists may boost the therapeutic efficacy of immune checkpoint inhibitors (ICIs) or molecularly targeted therapies in patients with HCC (9, 10). However, the mechanisms of chemokines in TIME are only just beginning to be discovered (5). In addition, studies of chemokines or chemokine receptors in HCC remain scarce, particularly in the prediction of survival in HCC patients and the relationship between the proportion of various immune cell subtypes in TIME and the expression of chemokines-related genes (CRGs).

In the present study, we first established and substantiated a kind of HCC predictive risk signature based on chemokine and chemokine receptor family genes. Nomograms were then developed and evaluated to precisely and conveniently forecast the outcome of HCC patients. Subsequently, the association between CRGs and immune infiltrating cells were analyzed at the transcriptomic and genomic mutational levels, respectively. Finally, we verified the differential expression of CRGs in HCC tissues and adjacent non-tumor tissue using real-time quantitative polymerase chain reaction (RT-qPCR).

## Materials and methods

### Data acquisition and processing

**Figure 1:**
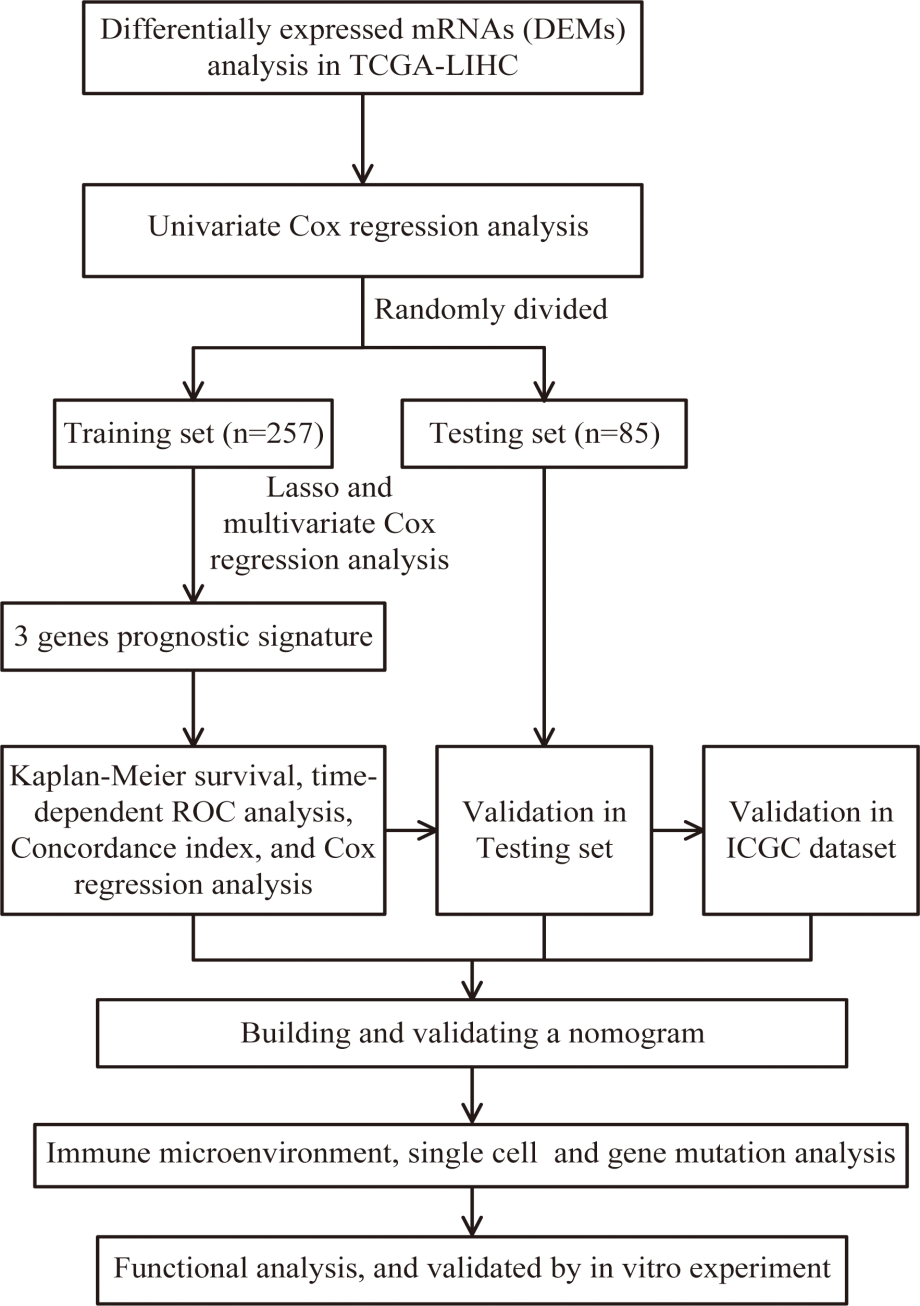
Flow chart of screening for chemokines- and chemokine receptors-based signature in hepatocellular carcinoma.

Download mRNA expression profiles and pathological features of HCC patients from The Cancer Genome Atlas (TCGA) and International Cancer Genome Consortium (ICGC) databases. 342 HCC patients were screened from the TCGA-LIHC cohort, excluding patients with missing expression data and those with survival times of less than 30 days (n=35). 231 HCC patients were obtained from the ICGC-LIHC-JP cohort as an external validation dataset. **Table 1** summarizes the detailed clinical profiles of the HCC patients enrolled in this study. Gene expression data of normal liver tissue were downloaded from the GTEx portal. scRNA-seq data for GSE149614 were obtained from the Gene Expression Omnibus (GEO) database, including 10 cases of primary tumor (PT), 2 cases of portal vein tumor thrombus (PVTT), 1 case of metastatic lymph node (MLN) and 8 cases of non-tumor liver tissue (NTL). The single-cell data were filtered by setting each gene to be expressed in a minimum of 3 cells, with each cell expressing at least 250 genes, resulting in 71915 cells. The percentage of mitochondria and rRNA was calculated using the “PercentageFeatureSet” function and ensuring that each cell expressed >100 and less than 8000 genes, that the mitochondrial content was less than 10% and that the UMI of each cell was at least >100, resulting in 67101 cells. We then normalized the data for each of the 21 samples by log-normalization. Variable features were identified by finding highly variable genes (based on variance stabilization transformation using the “FindVariableFeatures” function. All genes were scaled using the “ScaleData” function. Then we removed batch effects between samples and integrated the data by using the “FindIntegrationAnchors” and “IntegrateData” functions. Subsequently, we performed PCA downscaling to find anchor points by “RunPCA” function, selecting a dim of 40, and clustering the cells by using the “FindNeighbors” and “FindClusters” functions (setting Resolution=3) to obtain a total of 51 subgroups. Meanwhile, we used the “RunTSNE” function to perform T-distributed Stochastic Neighbour Embedding (tSNE) downscaling analysis on 67101 cells. Cells were annotated through published literature, the cell-marker website, and the “SingleR” package. Malignant cells were predicted using the “copycat” package. The genes used for the final annotation are shown in **Figure S1**). The detailed workflow diagram and corresponding analysis for the study were illustrated in **Figure 1**.

**Table 1:** Clinical characteristics of HCC patients.

Characters	Training dataset n=257	Testing dataset n=85	ICGC dataset (LIRI-JP) n=231
Age			
≤ 65	164 (63.81%)	52 (61.18%)	89 (38.5%)
> 65	93 (%)	33 (38.82%)	142 (61.5%)
Gender			
Female	77 (29.96%)	32 (37.65%)	61 (26.4%)
Male	180 (70.04%)	53 (62.35%)	170 (73.6%)
Grade			
G1-G2	155 (60.31%)	59 (69.41%)	N/A
G3-G4	97 (37.74%)	26 (30.59%)	N/A
Unknow	5 (1.95%)	0 (0%)	N/A
TNM stage			
I-II	183 (71.21%)	55 (64.71%)	141 (61.0%)
III-IV	59 (22.96%)	24 (28.24%)	90 (39.0%)
Unknown	15 (5.84%)	6 (7.06%)	0 (0%)
Tumor stage			
T1-T2	194 (75.49%)	58 (68.24%)	N/A
T3-T4	60 (23.35%)	27 (31.76%)	N/A
Unknow	3 (1.17%)	0 (0%)	N/A
Survival status			
Alive	169 (65.8%)	50 (58.8%)	189 (81.8%)
Deceased	88 (34.2%)	35 (41.2%)	42 (18.2%)

N/A, Not available.

### Development of prognostic risk signature

A total of 66 CRGs, including 47 chemokine ligands and 19 different chemokine receptors, were included in this study based on previous literature reports (**Table S1**) (11). In the TCGA dataset, we used the R project “limma” package to screen differentially expressed CRGs between HCC and adjacent non-tumor tissue with thresholds set to false discovery rate (FDR)< 0.05 and |log2 fold-change (FC)| > 1. CRGs related to overall survival (OS) in HCC patients were screened using univariate Cox regression analysis with a screening criterion of P< 0.05. The 342 HCC patients were subsequently randomized in a 3:1 ratio into the training and testing group using the R project “caret” package. The least absolute shrinkage and selection operator (LASSO) analysis with one standard error (SE) and 1000-fold cross-validation to filter the most significant markers in the training dataset using the “glmnet” and “survival” R packages. Given the simplicity and repeatability of the model, a backward stepwise Cox proportional risk regression model was developed using multivariate Cox analysis. Determine the model with the lowest AIC value as the final model. The risk score for each HCC patient in the three cohorts was determined by the following formula:


$$Risk\;score=\sum_{i=1}^{n}[coefficient\left(Gene\;i\right)*expression\left(Gene\;i\right)$$

### Assessment and confirmation of the prognostic risk signature and nomogram

All HCC patients in the three cohorts were allocated into the high- and low-risk groups according to the median risk score. Kaplan-Meier analysis was utilized to assess and compare the differences in survival outcomes between high- and low-risk groups of HCC patients in the training, test and ICGC datasets, respectively. The nomogram was constructed using the ‘rms’ and ‘regplot’ R packages, combining tumor staging and risk scoring. Calibration and time-related receiver operating characteristic (time ROC) curves were used to assess the accuracy and discrimination of the risk model and the nomogram using the “time ROC” and “rms” R packages.

### The landscape of immune and gene mutation

The Cell-type Identification By Estimating Relative Subsets Of RNA Transcripts (CIBERSORT) algorithm was implied for the quantitative assessment of the transcriptomic data and subsequently translated into the abundance of 22 types of immune and stromal cells (12). The single sample GSEA (ssGSEA) approach was applied to evaluate the inflammatory infiltration profiles and immune functions. Furthermore, we evaluated various immune cell infiltration using different algorithms with the Tumor Immune Estimation Resource (TIMER) database (http://timer.comp-genomics.org/) (13, 14). The Somatic Copy Number Alterations (SCNA) module of the TIMER database was adopted to investigate the association between specific genes and immune infiltration. Gene somatic mutation data were downloaded from Genomic Data Commons (GDC) database. The “oncoplot” function in the “maftools” package was performed to plot the respective mutations in the high- and low-risk groups and estimated the significantly different distribution of mutated genes by the “mafCompare” function in the “maftools” package.

### Drug sensitivity assessment and enrichment analysis

The semi-inhibitory concentration (IC50) values of well-known chemotherapeutic and targeted therapeutic agents were predicted by the “oncoPredict” R package to compare their efficacy in the different risk groups (15). Hallmark gene set and KEGG analysis were conducted using Gene Set Enrichment Analysis (GSEA) software 4.1.0. Gene ontology (GO) analysis was performed employing the R package “clusterProfiler” for identifying the functional differences between the two groups (16, 17). Furthermore, the gene sets of “c2.cp.kegg.v7.4.symbols.gmt” were downloaded from the MSigDB database to run GSVA enrichment analysis using the “GSVA” R package (18).

### RT-qPCR and proteomics for further demonstration

A total of 19 paired HCC and adjacent non-tumor tissue specimens were collected from the First Medical Centre of the Chinese PLA General Hospital. All specimens were quickly placed in liquid nitrogen (-196˚C) for preservation after excision. RNA extraction was performed using TRIzol reagent (Invitrogen, California, USA) and complementary DNA (cDNA) was constructed using ReverTra Ace qPCR RT Kit (Toyobo, Japan). Similarly, the detection and amplification of the prognostic genes were conducted using SYBR^®^ Green Realtime PCR Master Mix (Toyobo, Japan) in the ABI Step One Plus Real-Time PCR system (Applied Biosystems) while β-Actin was kept as an endogenous control. Each sample was repeated three times. The primer sequences are given in **Table S2**. Differences in gene expression between HCC and its paired adjacent non-tumor tissues were compared using the 2^−ΔΔCt^ approach. In addition, in the CPTAC dataset (Clinical Proteomics), we analyzed the protein expression levels of CCL14 and CCL20 in HCC using the UALCAN portal.

### Statistical analysis

The Kruskal-Wallis test or the Mann-Whitney U test was used for comparisons of continuous variables. Categorical variables were indicated as frequencies and compared using either the chi-square test or Fisher’s exact test. Survival curves were created using the Kaplan-Meier method and compared by applying a log-rank test. Independent risk factors associated with OS were identified using univariate and multivariate Cox regression analysis. P values< 0.05 were statistically significant and all tests were two-tailed. Data analysis and processing using R version 4.1.0, SPSS 26.0 and GraphPad Prism 8.

## Results

### The construction of risk signature on chemokine-related genes

A total of 19 CRGs were sieved in the TCGA database that was differentially expressed between HCC and adjacent non-tumor tissues (**Figures S2A, B**). Four of the 19 differentially expressed genes were notably related to overall survival time in HCC patients (**Figure S2C**). In the training dataset, we selected the candidate with the lowest AIC value as the final risk model (**Figure S2D**). Ultimately, the risk score for each HCC patient was obtained by the following equation:


Risk score=CCR3*0.501+CCL20*0.089−CCL14*0.84

### Assessment and validation of prognostic risk signature

**Figure 2:**
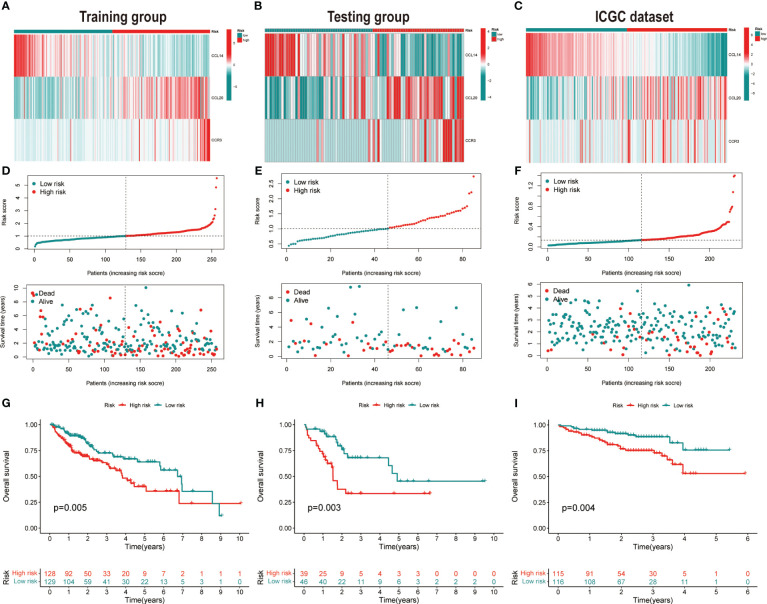
Validation of the prognostic signature. Heatmap of cluster analysis of HCC patients in **(A)** training, **(B)** testing, and **(C)** ICGC (LIRI-JP) dataset showing the expression levels of genes in the prognostic model. Distribution of risk score in the **(D)** training, **(E)** testing, and **(F)** ICGC (LIRI-JP) dataset, as well as survival time and survival condition of patients in the low-risk and high-risk groups. Kaplan-Meier survival analysis of chemokine-related gene prognostic signature in the **(G)** training, **(H)** testing, and **(I)** ICGC (LIRI-JP) dataset.

HCC patients were classified into two groups (high risk and low risk), depending on median risk values in the training, testing and ICGC dataset, respectively. There were no apparent discrepancies in clinical characteristics between high- and low-risk groups in the TCGA cohort, while the tumor stage and risk score were significantly correlated in the ICGC cohort (**Table 2**). In the three cohorts, risk scores decreased with the increased expression of CCL14 and increased when the expression of CCR3 and CCL20 increased (**Figures 2A–C**). Mortality in HCC patients tends to increase with higher risk score in the training (**Figure 2D**), testing (**Figure 2E**), and ICGC dataset (**Figure 2F**). Furthermore, HCC patients in the low-risk group had better overall survival outcomes compared to those in the high-risk group. (P< 0.05). (**Figures 2G–I**). The results of univariate (**Figures S3A, B**) and multivariate (**Figures S3C, D**) Cox regression analyses indicated that the risk score was an independent high-risk factor for HCC patients (P< 0.05). The time-dependent ROC curve (time ROC) analysis was further employed to assess the credibility and accuracy of the signature. The AUC of time ROC for this model to predict one, two, and three years OS in HCC patients was 0.683, 0.627, and 0.615 in the training dataset. (**Figure S4A**). Correspondingly, the AUC values for predicting one, two, three years OS in HCC patients were 0.719, 0.720, 0.656 (**Figure S4B**) and 0.644, 0.663, 0.637 (**Figure S4C**) in the testing and external validation datasets respectively. The results of Harrell’s concordance index (C-index) for the risk model and clinical features to predict one-five years OS in three groups were shown in **Figures S4D-F**.

**Table 2:** Baseline characteristics of HCC patients in the high- and low-risk groups.

Characters	TCGA dataset		P value	ICGC dataset (LIRI-JP)		P value
	High risk	Low risk		High risk	Low risk	
Age			0.275			0.244
≤ 65	110	106		40	49	
> 65	56	69		75	67	
Gender			0.776			0.626
Female	52	57		32	29	
Male	115	118		83	87	
Grade			0.097			N/A
G1-G2	95	119		N/A	N/A	
G3-G4	72	56		N/A	N/A	
Unknown	2	3		N/A	N/A	
TNM stage			0.079			< 0.001
I-II	110	128		55	86	
III-IV	49	34		60	30	
Unknown	8	13		N/A	N/A	
Tumor stage			0.073			N/A
T1-T2	118	134		N/A	N/A	
T3-T4	49	38		N/A	N/A	
Unknown	0	3		N/A	N/A	

N/A, Not available.

### Construction and assessment of the nomogram

**Figure 3:**
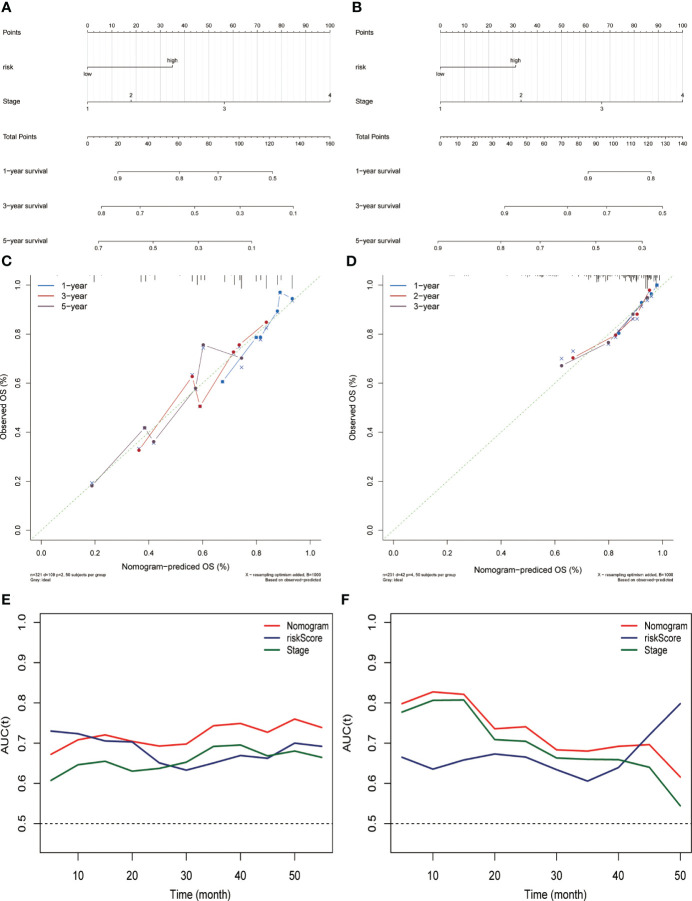
Constructing and evaluating the nomograms related to the risk score. Nomogram for predicting the probability of overall survival at 1, 3, and 5 years for HCC patients in the **(A)** TCGA dataset and **(B)** ICGC (LIRI-JP) dataset. Calibration plot for nomogram in the **(C)** TCGA dataset and **(D)** ICGC (LIRI-JP) dataset. Nomogram-based survival probabilities are plotted on the x-axis; actual survival rates are plotted on the y-axis. The time-dependent area under ROC curves of the nomogram, risk score, and stage for OS prediction in the **(E)** TCGA and **(F)** ICGC (LIRI-JP) datasets.

Combining risk score and tumor staging, we constructed nomograms in the TCGA (**Figure 3A**) and ICGC (**Figure 3B**) datasets, respectively. This can be applied as a clinically relevant quantitative method to enable clinicians to predict the survival outcome of HCC patients more accurately. The results of calibration plots indicated that the performance of the nomograms was comparable to the ideal model (**Figures 3C, D**). The time-dependent ROC of the two cohorts also demonstrated the high accuracy and predictive power of the nomograms (**Figures 3E, F**).

### Immune cell infiltration in the TCGA dataset based on risk score groupings

**Figure 4:**
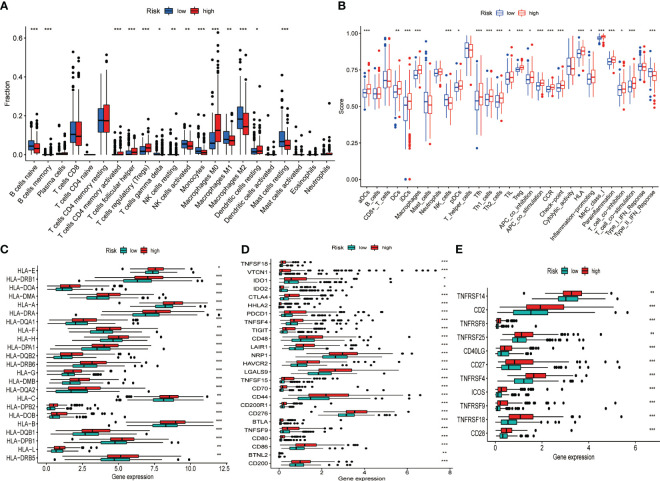
Differences in the TIME and the expression of immune-related gene sets between the different risk groups in the TCGA dataset. **(A)** Differences in the proportion of 22 immune cells infiltrating tumor tissue in the high- and low-risk groups. **(B)** Box plot depicting the difference in immune scores and immune function between the high- and low-risk patients of HCC. Gene expression of HLA **(C)**, immune checkpoint **(D)**, and T cell stimulators gene sets **(E)**. *P< 0.05, **P< 0.01, ***P< 0.001.

Given the intense relationship between CRGs and immune cells or immune function, we depicted and compared the discrepancies in the proportion of immune cells in TIME and immune function profiles between high- and low-risk groups using diverse algorithms. The results of CIBERSORT method discovered that B cell (naïve and memory), natural killer (NK) cells (resting and activated), monocytes, macrophages (M1, M2), and resting mast cells accounted for a substantially greater proportion of TIME in low-risk HCC than in high-risk group (P< 0.05). Activated memory CD4^+^ T cells, T-follicular helper (Tfh) cells, regulatory T cells (Tregs), M0 macrophages, and resting dendritic cells accounted for a significantly higher proportion of TIME in the high-risk HCC than in low-risk patients (P< 0.05) (**Figure 4A**). An overview of the 22 subtypes of tumor-infiltrating immune cells in the two groups is presented in **Figure S5A**. In addition, we calculated the Spearman correlation coefficients between each gene in the signature and 22 types of immune cells. The expression of CCL14 correlated negatively with M0 macrophages, Tregs, and memory B cells and positively with monocytes, M2 macrophages, NK cells and naive B cells (P< 0.05) (**Figure S5B**). The expression of CCL20 (**Figure S5C**) and CCR3 (**Figure S5D**) was negatively correlated with NK cells, monocytes and M2 macrophages and positively correlated with Tregs, CD4^+^ memory T cells and M0 macrophages (P< 0.05). The correlation between risk score and the proportion of immune cell subtypes in TIME calculated by different algorithms were illustrated in **Figure S5E**. The single sample GSEA (ssGSEA) algorithm obtained similar findings for immune cell infiltration as the CIBERSORT algorithm. Furthermore, there were also considerable differences in immune functions between the two groups, with significant activation of immune checkpoints, human leukocyte antigen (HLA), pro-inflammation, para-inflammation, T cell co-stimulation, and CC motif chemokine receptor (CCR) in the high-risk group (**Figure 4B**). In addition, we found that the expression of HLA genes (**Figure 4C**), immune checkpoint genes (**Figure 4D**), and T-cell stimulation genes (**Figure 4E**) was significantly higher in the high-risk group.

### scRNA analysis of the CCL cellular pathway network in PT, PVTT and MLN

**Figure 5:**
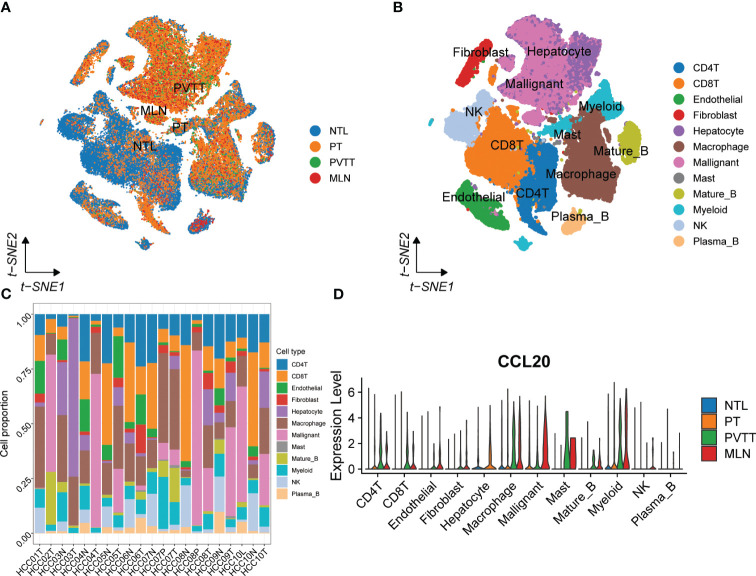
scRNA-seq analysis of primary and metastatic HCC and non-tumor liver tissue. **(A)** The tSNE map of 67101 cells from primary and metastatic HCC and paired non-tumor liver tissues. **(B)** Annotations for 12 cell types. **(C)** The proportion of different cell types in each sample. **(D)** The expression of CCL20 between different tissues and cell types.

**Figure 6:**
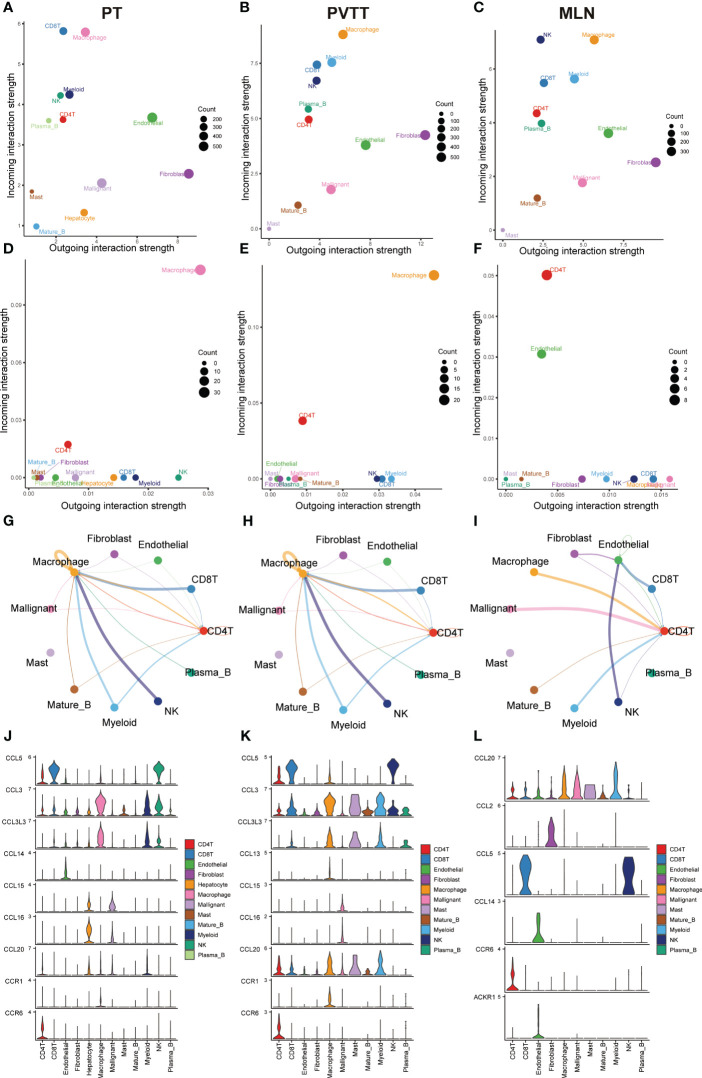
Results of cell-cell communication analysis in the CCL pathway. Bubble diagram visualizing the overall profile of possible incoming or outgoing signaling pathways between cells in **(A)** PT, **(B)** PVTT and **(C)** MLN. Bubble maps visualize possible incoming or outgoing CCL signaling pathways between cells in **(D)** PT, **(E)** PVTT and **(F)** MLN. The intensity of cell-cell interactions in the CCL signaling pathway in **(G)** primary tumor (PT), **(H)** portal vein tumor thrombosis (PVTT) and **(I)** metastatic lymph nodes (MLN). The expression of genes in the CCL signaling pathway in **(J)** PT, **(K)** PVTT and **(L)** MLN in different cell subpopulations.

To systematically assess the role of the CCL signaling pathway in the immune microenvironment and tumor progression of HCC, we analyzed scRNA-seq data. The results of the data pre-processing in detail are shown in **Figures S6A–C**. A total of 67101 cells were identified in four relevant tissue types: non-tumor liver (NTL), primary tumor (PT), portal vein tumor thrombus (PVTT) and metastatic lymph node (MLN) (**Figure 5A**). These cell clusters were then marked into 12 cell types based on specific genetic markers (**Figure 5B**). The proportion of the 12 cell types in each sample was shown in **Figure 5C**. We subsequently found that the expression of CCL20 was higher in CD4+ T cells, CD8+ T cells, endothelial cells, macrophages, malignant cells, mast cells, mature B cells, and myeloid cells in PVTT and MLN than in other cell and tissue types (**Figure 5D**). We investigated the overall profile (**Figures 6A–C**) and the CCL signaling pathway (**Figures 6D–F**) of differences in possible incoming or outgoing signaling pathways between different types of tissues. We found that in the CCL signaling pathway of PT or PVTT and MLN, the main cells for incoming and outgoing signals were CD4+ T cells and macrophages, respectively. We subsequently studied the differences in cell-cell communication networks in the CCL signaling pathway between different types of tissues by calculating the communication probabilities and obtained concordant results (**Figures 6G–I**). Finally, we examined the expression of CCL signaling pathway-related genes in different cell subpopulations in three tissue types (**Figures 6J–L**). Interestingly, we observed that the expression of CCL20 dominated intercellular communication in MLN tissues and that CCL20 was highly expressed in macrophages, malignant cells and myeloid cells.

### Genetic variation analysis in the TCGA dataset

**Figure 7:**
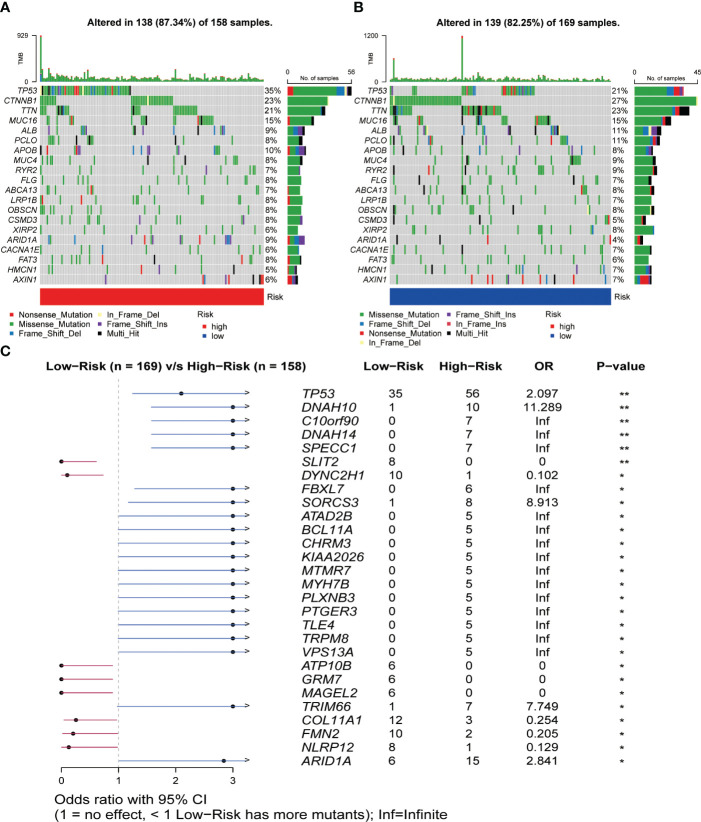
Relationship between somatic mutations and risk score in HCC. **(A)** Somatic mutations in the high-risk group. **(B)** Somatic mutations in the low-risk group. **(C)** Comparison of mutations between the high- and low-risk groups. *P< 0.05, **P< 0.01.

The results of the gene mutation analysis showed notable differences between the high-risk and low-risk groups of HCC patients, and we ranked the top 20 mutated genes (**Figures 7A, B**). However, we did not identify the mutations associated with CRGs. We also observed a higher incidence of mutations in common genes among the high-risk group, compared to the low-risk group of HCC patients. Furthermore, we identified a much higher frequency of mutations in TP53, ARID1A, DNAH10, and C10orf90 genes associated with tumorigenesis and progression in the high-risk group than in the low-risk group (**Figure 7C**). Subsequently, we initially investigated the type and frequency of alterations among chemokine family genes in HCC samples (**Figure S7A**), as well as the specific location of these genes in the chromosomes (**Figure S7B**). The relationship between the level of tumor immune infiltration and CNAs was further assessed using the TIMER database. The results suggested that changes in CCL14 and CCL20 somatic copy number alterations were closely associated with the level of immune cell infiltration (**Figure S8**).

### Risk score-based strategies for the treatment of HCC

**Figure 8:**
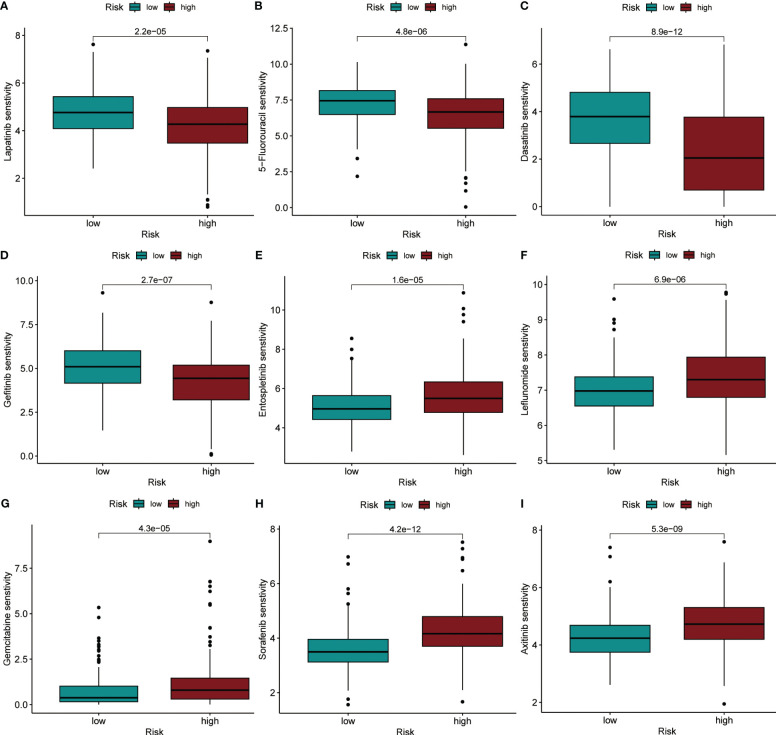
IC50 values were predicted for HCC patients between the different risk groups when treated with specific drugs. **(A)** Lapatinib. **(B)** 5-Fluorouracil. **(C)** Dasatinib. **(D)** Gefitinib. **(E)** Entospletinib. **(F)** Leflunomide. **(G)** Gemcitabine. **(H)** Sorafenib. **(I)** Axitinib.

Using the “oncoPredict” method, we calculated IC50 values for each HCC patient receiving chemotherapy or small molecule targeted therapy to predict their sensitivity when given different treatments. By comparison of the differences in IC50 values between different groups in the TCGA dataset, we identified that HCC patients in the high-risk group were more sensitive to treatment with Lapatinib, 5-Fluorouracil, Dasatinib, and Gefitinib (P< 0.001), compared to patients in the low-risk group (**Figures 8A–D**). Patients in the low-risk group were more sensitive to treatment with Entospletinib, Leflunomide, Gemcitabine, Sorafenib, and Axitinib, (P< 0.001) compared to patients in the high-risk group (**Figures 8E–I**).

### Functional analysis, RT-qPCR, and proteomics verification

**Figure 9:**
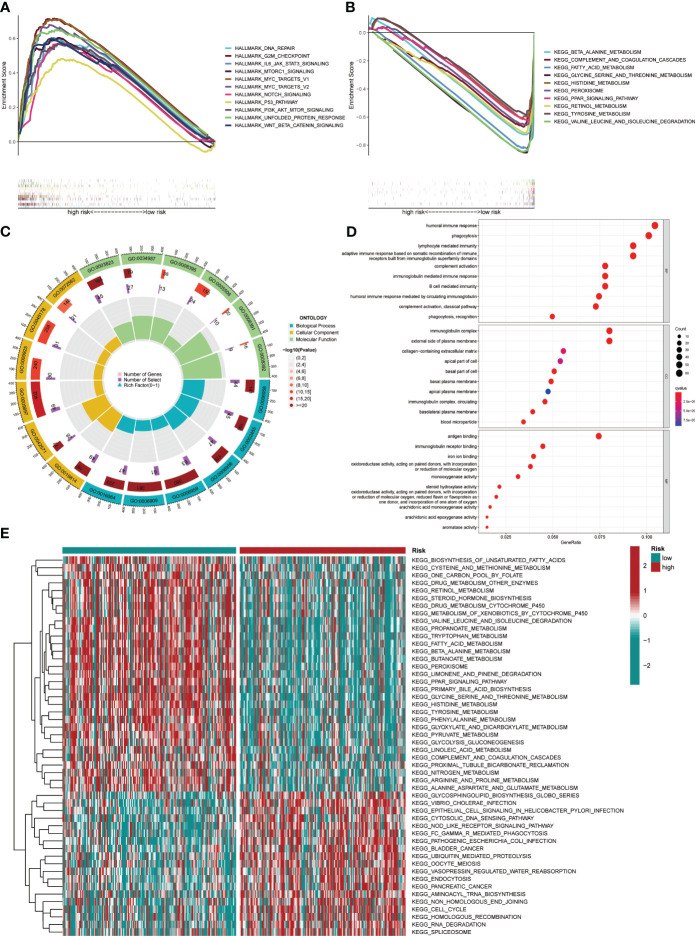
Pathway enrichment in the different risk groups and the expression of various gene sets between the two groups. **(A)** Gene set enrichment analysis (GSEA) of the Hallmark gene set for the high-risk group versus the low-risk group. **(B)** GSEA of KEGG pathway analysis for the low-risk group versus the high-risk group. **(C)** GO analysis indicated differentially enriched sets of genes between high- and low-risk groups. **(D)** Top 10 BP, CC, and MF terms in GO analysis. **(E)** Gene Set Variation Analysis.

**Figure 10:**
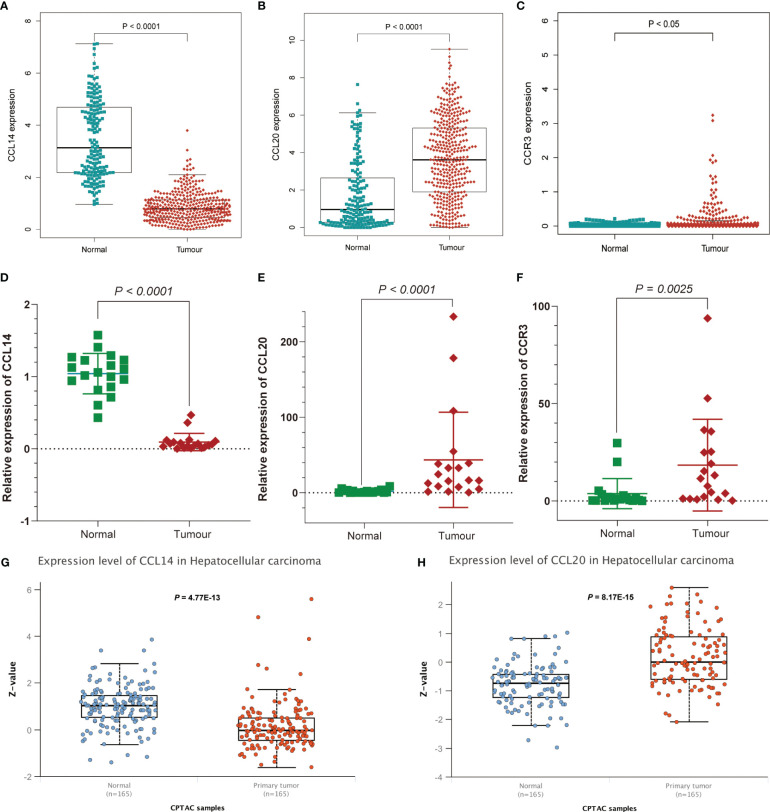
Further affirmation of the expression of genes in the signature. Analysis of **(A)** CCL14, **(B)** CCL20, and **(C)** CCR3 expression differences between HCC and normal tissues by combining TCGA and Genotype-Tissue Expression (GTEx) databases. Further demonstration of significant differences in the expression of **(D)** CCL14, **(E)** CCL20, and **(F)** CCR3 between HCC and normal tissues by RT-qPCR. Differential expression of **(G)** CCL14 and **(H)** CCL20 in normal and tumor tissues analyzed by UALCAN.

GSEA and GO functional analysis were carried out to delineate the differential gene expression profiles and function deviations between two risk groups, while GSVA analysis was conducted to elucidate the correlation between biological activity pathways and risk score. GSEA results of the hallmark genes set indicated that remarkable enrichment of immune-related pathways in the high-risk group compared to the low-risk group, such as IL6-JAK-STAT3, G2M-checkpoint, and P53 pathway (**Figure 9A**). On the other hand, the low-risk group was significantly enriched in metabolic function pathways compared to the high-risk group, such as β-alanine metabolism, fatty acid metabolism, and tyrosine metabolism (**Figure 9B**). All results of the GO functional analysis are shown in **Figure 9C** and **Table S3**. The main components of the bioprocess (BP) module are the humoral immune response, lymphocyte-mediated immunity, immunoglobulin-mediated immune response and other immune functions. The cellular component (CC) consists mainly of the immunoglobulin complex, the external side of the plasma membrane, and the collagen-containing extracellular matrix. The molecular Function (MF) primarily included antigen binding and immunoglobulin receptor binding (**Figure 9D**). A total of 121 enriched pathways were recognized by GSVA analysis among the two groups. The low-risk group primarily enriched in metabolism-associated pathways, while the high-risk group was primarily enriched in immune-related pathways (**Figure 9E** and **Table S4**). Given the paucity of adjacent non-tumor tissue samples in the TCGA database, we increased adjacent non-tumor tissue samples through the GTEx database and found that CCL14 (**Figure 10A**) was significantly highly expressed in adjacent non-tumor tissue, while CCL20 (**Figure 10B**) and CCR3 (**Figure 10C**) were significantly less expressed when compared with HCC tissue.

For the further investigation of the expression of the three genes (CCR3, CCL14, CCL20) in HCC tissues, RT-qPCR was applied to the detection of mRNA expression of the three genes between HCC tissues and adjacent non-tumor tissues. We affirmed the expression of genes in the signature and obtained consistent results (**Figures 10D–F** and **Table S5**). As for the expression of protein levels, the CPTAC database results showed that the expression of CCL14 in HCC tissues was significantly lower than that in normal tissues (**Figure 10G**), while the CCL20 expression level was significantly higher than that in normal tissues (**Figure 10H**).

## Discussion

Sophisticated genetic mutations and cellular dysfunction drive the formulation of hepatocellular carcinoma (HCC) with extensive heterogeneity. Compared to wide-spectrum molecular drive therapies that apply to specific patient populations for specific cancer types, current immunotherapy and targeted therapies for advanced HCC are “one-size-fits-all”, without detailed patient stratification, which limits the efficacy of systemic treatment and fueling the unsatisfied medical needs of HCC (19). The development of a novel, reliable prognostic scoring system is urgent to improve the risk stratification of HCC patients and predict the efficacy of systemic therapy (20). The cancer cell and tumor microenvironment could modulate the expression and function of chemokine-related genes, which in turn shapes the type and consequence of the immune response to malignant cells and cancer systemic therapy (5). However, no studies are focusing on the relationship between CRGs and the prognosis of HCC patients or the tumor immune microenvironment.

In this study, we have established a prognostic signature based on chemokines and chemokine receptors. This signature showed strong predictive power for HCC patients with an external dataset. Notably, we have also developed and evaluated nomograms, which have good accuracy and reliability to facilitate clinical application. The comprehensive multi-omics analysis could detect and discover not only the meticulous genetic modifications and mutations in HCC but also the precise composition of the different cell types in the TIME and their interactions with hepatocellular carcinoma cells (21, 22). Therefore, we investigated the association between the chemokine-based signature and the various subtypes of immune cells in the TIME employing different algorithms. We further demonstrated that chemokines play an integral part in the shaping of different types of TIME, which have significant implications for tumor progression and treatment outcome in the TCGA cohort.

We identified two chemokine ligands and one chemokine receptor, CCL14, CCL20 and CCR3, which are uniquely advantageous in predicting the prognosis of HCC patients. It has been shown that the overexpression of CCL14 inhibits cancer cell proliferation and promotes apoptosis and that its low expression is associated with poor prognosis in HCC patients (23). MMP-21 promotes macrophage recruitment by increasing CCL-14 levels and M2 macrophage polarization by increasing CSF-1 and FGF-1 expressions, thereby regulating the immune microenvironment and metastasis of HCC (24). The CCL20-CCR6 axis has been reported to be associated with a variety of cancers, including HCC, colorectal cancer, breast cancer, pancreatic cancer, cervical cancer and renal cancer (25). The CCL20-CCR6 axis facilitates cancer progression directly by potentiating the migration and proliferation of cancer cells, and indirectly by reshaping the tumor microenvironment through immune cell manipulation (25). Consistent with our single-cell analysis results, CCL20 gene expression was significantly higher in metastatic HCC tissues (PVTT and MLN). Moreover, the CCL20 gene dominates intercellular communication in MLN and was highly expressed in macrophages, malignant cells and myeloid cells. There is also a study using online databases that have found significantly higher expression of CCL20 in HCC tissues compared to the adjacent non-tumor tissues (26). Another retrospective clinical study revealed that the expression of CCL20 was significantly associated with tumor recurrence and survival outcomes in HCC patients (27). These results were consistent with our study. In addition, the role of CCL20 in the TIME is manifested mainly through the recruitment of Th17 cells, immature DCs, Tregs and tumor-associated macrophages (TAMs) (5). No studies of CCR3 in HCC have been reported. However, studies in other types of cancer have shown that CCR3 plays a major role in promoting tumor progression and is strongly associated with poor prognosis of patients, with its role in the TIME exhibited through the recruitment of TAMs to facilitate tumor progression (5, 28–30). In addition, it has been found that inhibition of CCR3 in cancer cell lines induces polyploid giant cell formation and β-catenin stabilization through the PI3K/Akt/GSK-3β signaling pathway, a process associated with EMT, as a result of CCR3 inhibition, transformed cells acquired enhanced mobility and proliferation (31).

Non-polarized (M0) macrophages are derived from monocytes following colony-stimulating factor 1 (CSF-1) induction. M0 macrophages can be differentiated into M1 and M2 macrophages when stimulated by different cytokines. M1 macrophages exhibited cytotoxic properties on tumors by secreting cytokines such as IL-2 and TNF-α. In contrast, M2 macrophages have the potential to promote tumor progression (32). Furthermore, the increased infiltration of TAMs in TIME was associated with worse patient outcomes based on TCGA analysis of HCC (33). The phenotypes of dendritic cells were complex, with either subtype that promotes CD8+ T cell activation or subtypes that contribute to T cell dysfunction (33). Tfh cells were known to promote anti-tumor immune responses but suffered strong immunosuppression due to the high expression of PD-1 (34). NK cells are a subtype of immune cells with anti-tumor properties and serve an indispensable part in the immune surveillance and eradication of tumors. However, in the environment of tumor or chronic infection, NK cells manifest a state of exhaustion similar to that of T cell exhaustion, with depressed effector function and altered phenotype (35). Tregs are a subset of lymphocytes with highly immunosuppressive properties that suppress tumor-infiltrating cytotoxic T lymphocytes and accumulate commonly in HCC (36). The increased infiltration of Tregs in TIME is firmly associated with the aggressiveness and progression of HCC, as well as poor patient prognosis (37). In the present research, macrophages, Tregs, dendritic cells, and Tfh cells accounted for significantly higher proportions of TIME in the high-risk group compared to the low-risk group. At the same time, NK cells, monocytes, and M1 macrophages comprised a substantially more proportion of TIME in the low-risk group. The findings could provide an explanation for the unfavorable outcomes of HCC patients in the high-risk group. In addition, we observed remarkable activation of immune-related functions such as HLA, T cell co-stimulation and immune checkpoints in the high-risk group of HCC patients. The activation of immune functions in these three groups was then confirmed at the level of gene expression. These results corresponded to a higher frequency TMB in the high-risk group of HCC patients. Since meaningful mutations occurring in tumors are tailored into neoantigens which are delivered to CD8+ T cells *via* major histocompatibility complex (MHC) proteins. The increased TMB results in larger numbers of neoantigens, increasing the opportunity for recognition by CD8+ T cells (38).

We also stratified HCC patients in the TCGA dataset according to risk score profile and assessed the sensitive properties of patients in different groups to specific drugs. Sorafenib was the first small molecule-targeted drug to be demonstrated effectiveness in the systemic therapy of advanced HCC and remains one of the first-line of treatments for advanced HCC (39). Our prediction results indicated lower IC50 values in the low-risk group of HCC patients treated with sorafenib. This suggested that HCC patients in the high-risk group were not as sensitive to sorafenib as those in the low-risk group. In addition, we identified six small molecule-targeted drugs and three chemotherapeutic drugs with meaningful differences in IC50 values in the different risk groups, which shed light on potential chemotherapies and targeted therapy for HCC.

GSEA, GO, and GSVA analysis provides mechanistic insights into the discrepancy in the infiltration of various immune cell subtypes of TIME in different groups. GSEA profiling indicated that immune function-related signaling pathways such as Notch, MAPK, P53, and Jak-Stat were notably abundant in the high-risk group of the TCGA cohort. These pathways serve as critical components in the development of HCC and targeted therapy (40–43). Correspondingly, metabolism-related signaling pathways were substantially concentrated in the low-risk group, while interactions between nutrients, metabolites and immune cells played a principal role in immune editing and tumor escape. Metabolic reprogramming, for example, is the backbone of T cell differentiation and activation, as the transition from quiescence to activation, proliferation, differentiation and infiltration bears a heavy energy burden (44, 45). We obtained consistent results from GO and GSVA analyses, with considerable discrepancy in the enrichment of immune- and metabolism-associated signaling pathways in the two groups of HCC patients. All of the above results provide sufficient evidence for the immune or inflammatory activeness profile of chemokine-associated signaling in TIME for patients with HCC, and firmly substantiate the underlying mechanism by which the signature predicts the prognosis of HCC patients.

Although the chemokine-related signature is a valid independent prognostic factor and was strongly associated with TIME in patients with HCC, some limitations should still be recognized. Firstly, all cohorts used for analysis are sourced from the public database and the conclusions require additional external data validation. Second, the genes in the signature need to be further validated in cellular and animal models for understanding the functions and mechanisms involved in TIME and tumor development.

In conclusion, a novel signature was successfully developed and substantiated to forecast the prognostic outcome of patients with HCC. The relationship and potential mechanisms involved in the chemokines-related signature and the TIME in HCC were preliminarily explored. This study presents a promising landscape for clinical research in the application or targeting of chemokines in monotherapy or combination therapies.

## Data availability statement

Public data supporting the findings of this study are available from The Cancer Genome Atlas (TCGA) (https://cancergenome.nih.gov/), UCSC Xena (http://xena.ucsc.edu/), GTEx portal (http://www.gtexportal.org/home/), and ICGC database (https://dcc.icgc.org/).

## Ethics statement

The studies involving human participants were reviewed and approved by The ethics committee of the Chinese PLA General Hospital (Approval No. S2018-111-01). The patients/participants provided their written informed consent to participate in this study.

## Author contributions

ZZ, MM, FW, and ZPZ collected, analyzed and interpreted the data. FW, YZ, MM, JS, LC, and XW provide technical support and assistance with experiments. SL, PX, and ZZ conceived and designed the study. SL and ZZ drafted the manuscript. SL, PX, and ZPZ supervised research and manuscripts. All authors have read and approved the final version of the manuscript.

## Supplementary material

Supplementary Table 1 The chemokines and chemokine receptors are included in this study.

Supplementary Table 3 The results of Gene Ontology analysis.

Supplementary Table 4 Gene Set Variation Analysis.

Supplementary Table 5 RT-qPCR.

Supplementary Figure 1 Dot plot of the expression of marker genes used for cell annotation.

Supplementary Figure 2 Establishment of prognostic models based on CRGs in the training dataset. **(A)** Volcano plot of differentially expressed CRGs in HCC and normal tissues from TCGA dataset. **(B)** Heatmap of significant differentially expressed DECRGs. **(C)** Forest plot of the results of the Univariate Cox regression analysis. **(D)** Forest plot showing 3 DECRGs included in a multivariate Cox proportional hazards model to predict survival in HCC patients. The concordance of the risk model was 0.65, indicating a favorable predictive power.

Supplementary Figure 3 Identify independent prognostic factors in the TCGA dataset. Forest plots indicate 95% confidence intervals (95% CI) for hazard ratios (HRs) and p-values for multivariate Cox regression in the **(A)** training and **(B)** testing datasets.

Supplementary Figure 4 Assessment of the accuracy of risk score and clinical characteristics in predicting the prognosis of patients with HCC. **(A–C)** ROC curve analysis for the training group, testing group, and external validation group respectively. **(D, E)** Concordance index analysis for the training group, testing group, and external validation group respectively.

Supplementary Figure 5 Analysis of immune cell infiltration about risk score and prognostic genes in the TCGA database. **(A)** Overview of 22 immune cell infiltrations in the high- and low-risk groups. Correlation analysis of **(B)** CCL14, **(C)** CCL20, and **(D)** CCR3 with immune cells. **(E)** The landscape of immune cells infiltration between high- and low-risk groups by different algorithms.

Supplementary Figure 6 The results of scRNA-seq data reprocessing. **(A)** Relationship between mitochondrial genes and UMI/mRNA amount, UMI and mRNA amount. (B-upper) Relationship between mRNA, UMI, mitochondrial content and rRNA content of each sample before filtering; (B-lower) Relationship between mRNA, UMI, mitochondrial content and rRNA content of each sample after filtering. **(D)** Principal component downscaled sample distribution and principal component anchor point plots.

Supplementary Figure 7 The alterations of CRGs in the TCGA-LIHC cohort. **(A)** The proportions of copy number variations (CNVs) of CRGs in HCC. **(B)** Determining the chromosomal location of CRGs that occur CNVs in HCC.

Supplementary Figure 8 Relationship between the CNVs types of prognostic genes and immune cell infiltration in the TCGA database. *P< 0.05, ***P< 0.001.

## References

[1] Sung H Ferlay J Siegel RL Laversanne M Soerjomataram I Jemal A. Global cancer statistics 2020: GLOBOCAN estimates of incidence and mortality worldwide for 36 cancers in 185 countries. CA Cancer J Clin (2021) 71(3):209–49. doi: 10.3322/caac.21660 33538338

[2] Vogel A Meyer T Sapisochin G Salem R Saborowski A. Hepatocellular carcinoma. Lancet (London England) (2022) 400(10360):1345–62. doi: 10.1016/S0140-6736(22)01200-4 36084663

[3] Griffith JW Sokol CL Luster AD. Chemokines and chemokine receptors: positioning cells for host defense and immunity. Annu Rev Immunol (2014) 32:659–702. doi: 10.1146/annurev-immunol-032713-120145 24655300

[4] Vicari AP Caux C. Chemokines in cancer. Cytokine Growth Factor Rev (2002) 13(2):143–54. doi: 10.1016/S1359-6101(01)00033-8 11900990

[5] Ozga AJ Chow MT Luster AD. Chemokines and the immune response to cancer. Immunity (2021) 54(5):859–74. doi: 10.1016/j.immuni.2021.01.012 PMC8434759 33838745

[6] Märkl F Huynh D Endres S Kobold S. Utilizing chemokines in cancer immunotherapy. Trends Cancer (2022) 8(8):670–82. doi: 10.1016/j.trecan.2022.04.001 35501268

[7] Lazennec G Richmond A. Chemokines and chemokine receptors: new insights into cancer-related inflammation. Trends Mol Med (2010) 16(3):133–44. doi: 10.1016/j.molmed.2010.01.003 PMC2840699 20163989

[8] Mollica Poeta V Massara M Capucetti A Bonecchi R. Chemokines and chemokine receptors: new targets for cancer immunotherapy. Front Immunol (2019) 10:379. doi: 10.3389/fimmu.2019.00379 30894861 PMC6414456

[9] Yao W Ba Q Li X Li H Zhang S Yuan Y. A natural CCR2 antagonist relieves tumor-associated macrophage-mediated immunosuppression to produce a therapeutic effect for liver cancer. EBioMedicine (2017) 22:58–67. doi: 10.1016/j.ebiom.2017.07.014 28754304 PMC5552238

[10] Li X Yao W Yuan Y Chen P Li B Li J. Targeting of tumor-infiltrating macrophages *via* CCL2/CCR2 signalling as a therapeutic strategy against hepatocellular carcinoma. Gut (2017) 66(1):157–67. doi: 10.1136/gutjnl-2015-310514 26452628

[11] Nagarsheth N Wicha MS Zou W. Chemokines in the cancer microenvironment and their relevance in cancer immunotherapy. Nat Rev Immunol (2017) 17(9):559–72. doi: 10.1038/nri.2017.49 PMC5731833 28555670

[12] Newman AM Liu CL Green MR Gentles AJ Feng W Xu Y. Robust enumeration of cell subsets from tissue expression profiles. Nat Methods (2015) 12(5):453–7. doi: 10.1038/nmeth.3337 PMC4739640 25822800

[13] Li T Fu J Zeng Z Cohen D Li J Chen Q. TIMER2.0 for analysis of tumor-infiltrating immune cells. Nucleic Acids Res (2020) 48(W1):W509–w514. doi: 10.1093/nar/gkaa407 32442275 PMC7319575

[14] Li T Fan J Wang B Traugh N Chen Q Liu JS. TIMER: a web server for comprehensive analysis of tumor-infiltrating immune cells. Cancer Res (2017) 77(21):e108–10. doi: 10.1158/1538-7445.AM2017-108 PMC6042652 29092952

[15] Maeser D Gruener RF Huang RS. oncoPredict: an r package for predicting. Vivo Cancer patient Drug response Biomarkers Cell line screening data Briefings In Bioinf (2021) 22(6). doi: 10.1093/bib/bbab260 PMC8574972 34260682

[16] Drabkin HJ Hill DP Carbon S Dietze H Mungall CJ Munoz-Torres MC. Gene ontology consortium: going forward. Nucleic Acids Res (2015) 43(Database issue):D1049–1056. doi: 10.1093/nar/gku1179 PMC4383973 25428369

[17] Yu G Wang L-G Han Y He Q-Y. clusterProfiler: an r package for comparing biological themes among gene clusters. Omics J Integr Biol (2012) 16(5):284–7. doi: 10.1089/omi.2011.0118 PMC3339379 22455463

[18] Liberzon A Birger C Thorvaldsdóttir H Ghandi M Mesirov JP Tamayo P. The molecular signatures database (MSigDB) hallmark gene set collection. Cell Syst (2015) 1(6):417–25. doi: 10.1016/j.cels.2015.12.004 PMC4707969 26771021

[19] Chan L-K Tsui Y-M Ho DW-H Ng IO-L. Cellular heterogeneity and plasticity in liver cancer. Semin Cancer Biol (2022) 82:134–49. doi: 10.1016/j.semcancer.2021.02.015 33647386

[20] Calderaro J Seraphin TP Luedde T Simon TG. Artificial intelligence for the prevention and clinical management of hepatocellular carcinoma. J Hepatol (2022) 76(6):1348–61. doi: 10.1016/j.jhep.2022.01.014 PMC9126418 35589255

[21] Liu S Yang Z Li G Li C Luo Y Gong Q. Multi-omics analysis of primary cell culture models reveals genetic and epigenetic basis of intratumoral phenotypic diversity. Genomics Proteomics Bioinf (2019) 17(6):576–89. doi: 10.1016/j.gpb.2018.07.008 PMC7212478 32205176

[22] Kurebayashi Y Ojima H Tsujikawa H Kubota N Maehara J Abe Y. Landscape of immune microenvironment in hepatocellular carcinoma and its additional impact on histological and molecular classification. Hepatol (Baltimore Md) (2018) 68(3):1025–41. doi: 10.1002/hep.29904 29603348

[23] Zhu M Xu W Wei C Huang J Xu J Zhang Y. CCL14 serves as a novel prognostic factor and tumor suppressor of HCC by modulating cell cycle and promoting apoptosis. Cell Death Dis (2019) 10(11):796. doi: 10.1038/s41419-019-1966-6 31641099 PMC6805940

[24] Zhou J Liu L Hu X Feng R Zhao N Zhang L. Matrix metalloproteinase-21 promotes metastasis. via increasing recruitment M2 polarization macrophages HCC. Cancer Sci (2023) 114(2):423–35. doi: 10.1111/cas.15368 PMC9899621 35398966

[25] Kadomoto S Izumi K Mizokami A. The CCL20-CCR6 axis in cancer progression. Int J Mol Sci (2020) 21(15):5186. doi: 10.3390/ijms21155186 32707869 PMC7432448

[26] Jiang Z Xing C Wang P Liu X Zhong L. Identification of therapeutic targets and prognostic biomarkers among chemokine (C-c motif) ligands in the liver hepatocellular carcinoma microenvironment. Front Cell Dev Biol (2021) 9:748269. doi: 10.3389/fcell.2021.748269 34938730 PMC8685337

[27] Ding X Wang K Wang H Zhang G Liu Y Yang Q. High expression of CCL20 is associated with poor prognosis in patients with hepatocellular carcinoma after curative resection. J Gastrointestinal Surg Off J Soc For Surg Alimentary Tract (2012) 16(4):828–36. doi: 10.1007/s11605-011-1775-4 22072303

[28] Jöhrer K Zelle-Rieser C Perathoner A Moser P Hager M Ramoner R. Up-regulation of functional chemokine receptor CCR3 in human renal cell carcinoma. Clin Cancer Res an Off J Am Assoc For Cancer Res (2005) 11(7):2459–65. doi: 10.1158/1078-0432.CCR-04-0405 15814620

[29] Yamaguchi M Takagi K Narita K Miki Y Onodera Y Miyashita M. Stromal CCL5 promotes breast cancer progression by interacting with CCR3 in tumor cells. Int J Mol Sci (2021) 22(4):1918. doi: 10.3390/ijms22041918 33671956 PMC7919043

[30] Wang C Wang Y Hong T Cheng B Gan S Chen L. Blocking the autocrine regulatory loop of Gankyrin/STAT3/CCL24/CCR3 impairs the progression and pazopanib resistance of clear cell renal cell carcinoma. Cell Death Dis (2020) 11(2):117. doi: 10.1038/s41419-020-2306-6 32051393 PMC7015941

[31] Kaibori Y Nagakubo D. CCR3 blockage elicits polyploidization associated with the signatures of epithelial-mesenchymal transition in carcinoma cell lines. Cancer Gene Ther (2023) 30(1):137–48. doi: 10.1038/s41417-022-00532-8 36123391

[32] Tiwari A Trivedi R Lin S-Y. Tumor microenvironment: barrier or opportunity towards effective cancer therapy. J BioMed Sci (2022) 29(1):83. doi: 10.1186/s12929-022-00866-3 36253762 PMC9575280

[33] Zhang Q He Y Luo N Patel SJ Han Y Gao R. Landscape and dynamics of single immune cells in hepatocellular carcinoma. Cell (2019) 179(4):829–45.e820. doi: 10.1016/j.cell.2019.10.003 31675496

[34] Ma Q-Y Huang D-Y Zhang H-J Chen J Miller W Chen X-F. Function of follicular helper T cell is impaired and correlates with survival time in non-small cell lung cancer. Int Immunopharmacol (2016) 41:1–7. doi: 10.1016/j.intimp.2016.10.014 27788370

[35] Bi J Tian Z. NK cell exhaustion. Front Immunol (2017) 8:760. doi: 10.3389/fimmu.2017.00760 28702032 PMC5487399

[36] Gao Q Qiu S-J Fan J Zhou J Wang X-Y Xiao Y-S. Intratumoral balance of regulatory and cytotoxic T cells is associated with prognosis of hepatocellular carcinoma after resection. J Clin Oncol Off J Am Soc Clin Oncol (2007) 25(18):2586–93. doi: 10.1200/JCO.2006.09.4565 17577038

[37] Fu J Xu D Liu Z Shi M Zhao P Fu B. Increased regulatory T cells correlate with CD8 T-cell impairment and poor survival in hepatocellular carcinoma patients. Gastroenterology (2007) 132(7):2328–39. doi: 10.1053/j.gastro.2007.03.102 17570208

[38] Jardim DL Goodman A de Melo Gagliato D Kurzrock R. The challenges of tumor mutational burden as an immunotherapy biomarker. Cancer Cell (2021) 39(2):154–73. doi: 10.1016/j.ccell.2020.10.001 PMC7878292 33125859

[39] Llovet JM Ricci S Mazzaferro V Hilgard P Gane E Blanc J-F. Sorafenib in advanced hepatocellular carcinoma. N Engl J Med (2008) 359(4):378–90. doi: 10.1056/NEJMoa0708857 18650514

[40] Yang H Sun L Guan A Yin H Liu M Mao X. Unique TP53 neoantigen and the immune microenvironment in long-term survivors of hepatocellular carcinoma. Cancer Immunol Immunother (2021) 70(3):667–77. doi: 10.1007/s00262-020-02711-8 PMC10992148 32876735

[41] Calvisi DF Ladu S Gorden A Farina M Conner EA Lee J-S. Ubiquitous activation of ras and Jak/Stat pathways in human HCC. Gastroenterology (2006) 130(4):1117–28. doi: 10.1053/j.gastro.2006.01.006 16618406

[42] Majumder S Crabtree JS Golde TE Minter LM Osborne BA Miele L. Targeting notch in oncology: the path forward. Nat Rev Drug Discov (2021) 20(2):125–44. doi: 10.1038/s41573-020-00091-3 33293690

[43] Delire B Stärkel P. The Ras/MAPK pathway and hepatocarcinoma: pathogenesis and therapeutic implications. Eur J Clin Invest (2015) 45(6):609–23. doi: 10.1111/eci.12441 25832714

[44] Sugiura A Rathmell JC. Metabolic barriers to T cell function in tumors. J Immunol (Baltimore Md 1950) (2018) 200(2):400–7. doi: 10.4049/jimmunol.1701041 PMC5777533 29311381

[45] Zhang L Romero P. Metabolic control of CD8 T cell fate decisions and antitumor immunity. Trends Mol Med (2018) 24(1):30–48. doi: 10.1016/j.molmed.2017.11.005 29246759

