# Supplementary material for: Comprehensive analysis and immune landscape of chemokines- and chemokine receptors-based signature in hepatocellular carcinoma
Source: Front Immunol. 2023 Jul 20;14:1164669. doi: 10.3389/fimmu.2023.1164669 (PMC10399597; doi:10.3389/fimmu.2023.1164669)
Supplement: Supplementary file 2 [file Table_2.docx]

**Table S2. All the primer sequences of the article.**

**CCL20**  Forward: 5’-TGCTGTACCAAGAGTTTGCTC-3’

Reverse: 5’-CGCACACAGACAACTTTTTCTTT-3’;

**CCL14** Forward: 5’-GTGCTGCTTCACCTACACTACC-3’

Reverse: 5’-GATATAGTCCTGGACCCACTTGT-3’;

**CCR3** Forward: 5’-CTGTACTCCCTGGTGTTCACT-3’

Reverse: 5’-GTTGAGCAGGTAGATGTTGGT-3’;

**β-actin** Forward: 5’-TGACGTGGACATCCGCAAAG-3’

Reverse: 5’-CTGGAAGGTGGACAGCGAGG-3’.
