# Supplementary figures and images for: Comprehensive analysis and immune landscape of chemokines- and chemokine receptors-based signature in hepatocellular carcinoma
Source: Front Immunol. 2023 Jul 20;14:1164669. doi: 10.3389/fimmu.2023.1164669 (PMC10399597; doi:10.3389/fimmu.2023.1164669)

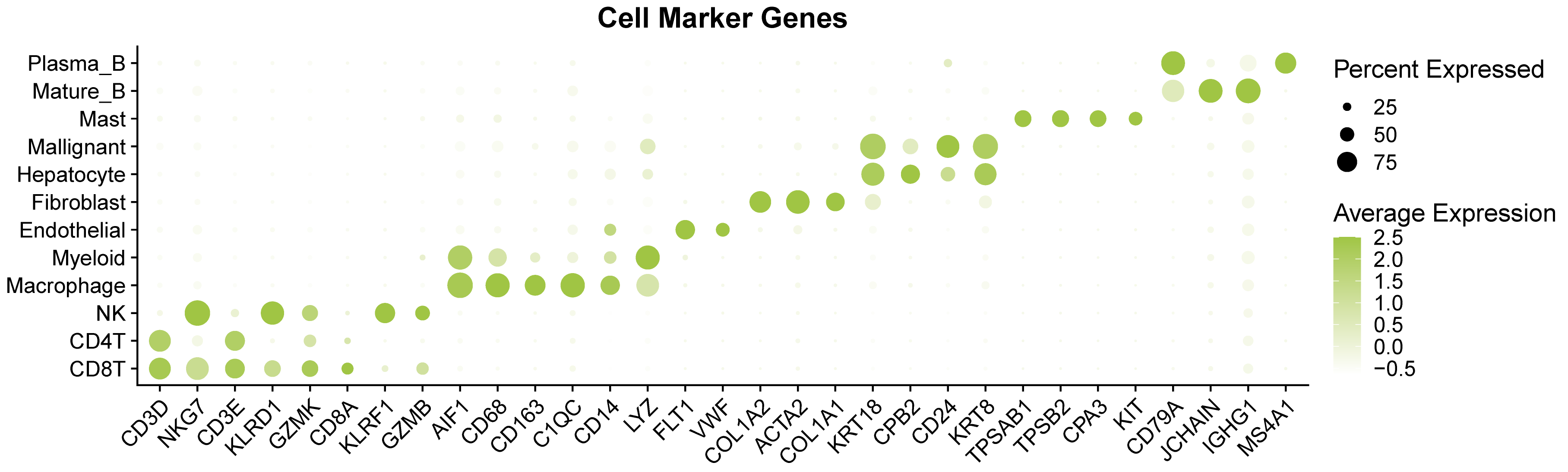

Supplement: Supplementary Figure 1 — Dot plot of the expression of marker genes used for cell annotation. [file Image_1.tif]

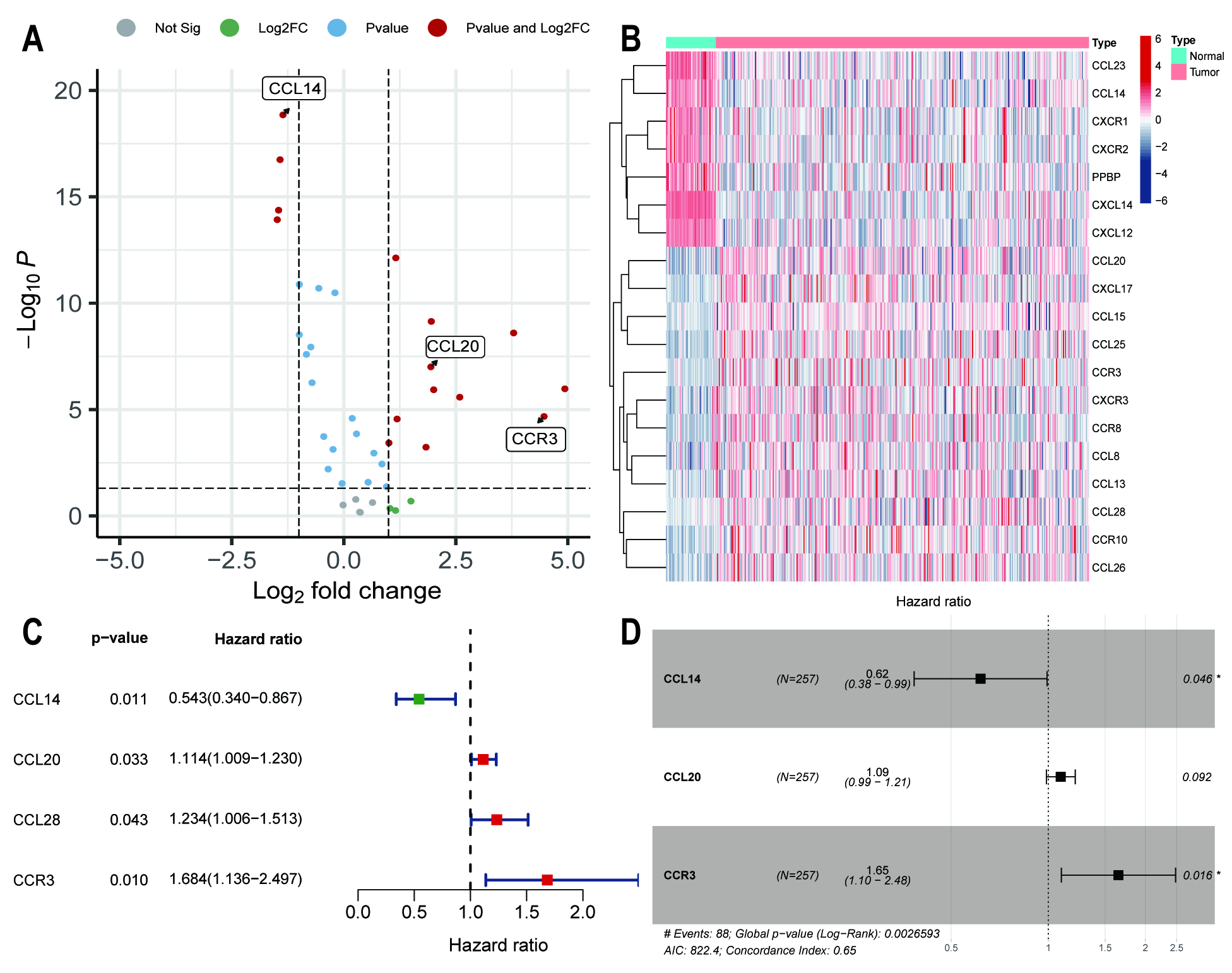

Supplement: Supplementary Figure 2 — Establishment of prognostic models based on CRGs in the training dataset. (A) Volcano plot of differentially expressed CRGs in HCC and normal tissues from TCGA dataset. (B) Heatmap of significant differentially expressed DECRGs. (C) Forest plot of the results of the Univariate Cox regression analysis. (D) Forest plot showing 3 DECRGs included in a multivariate Cox proportional hazards model to predict survival in HCC patients. The concordance of the risk model was 0.65, indicating a favorable predictive power. [file Image_2.tif]

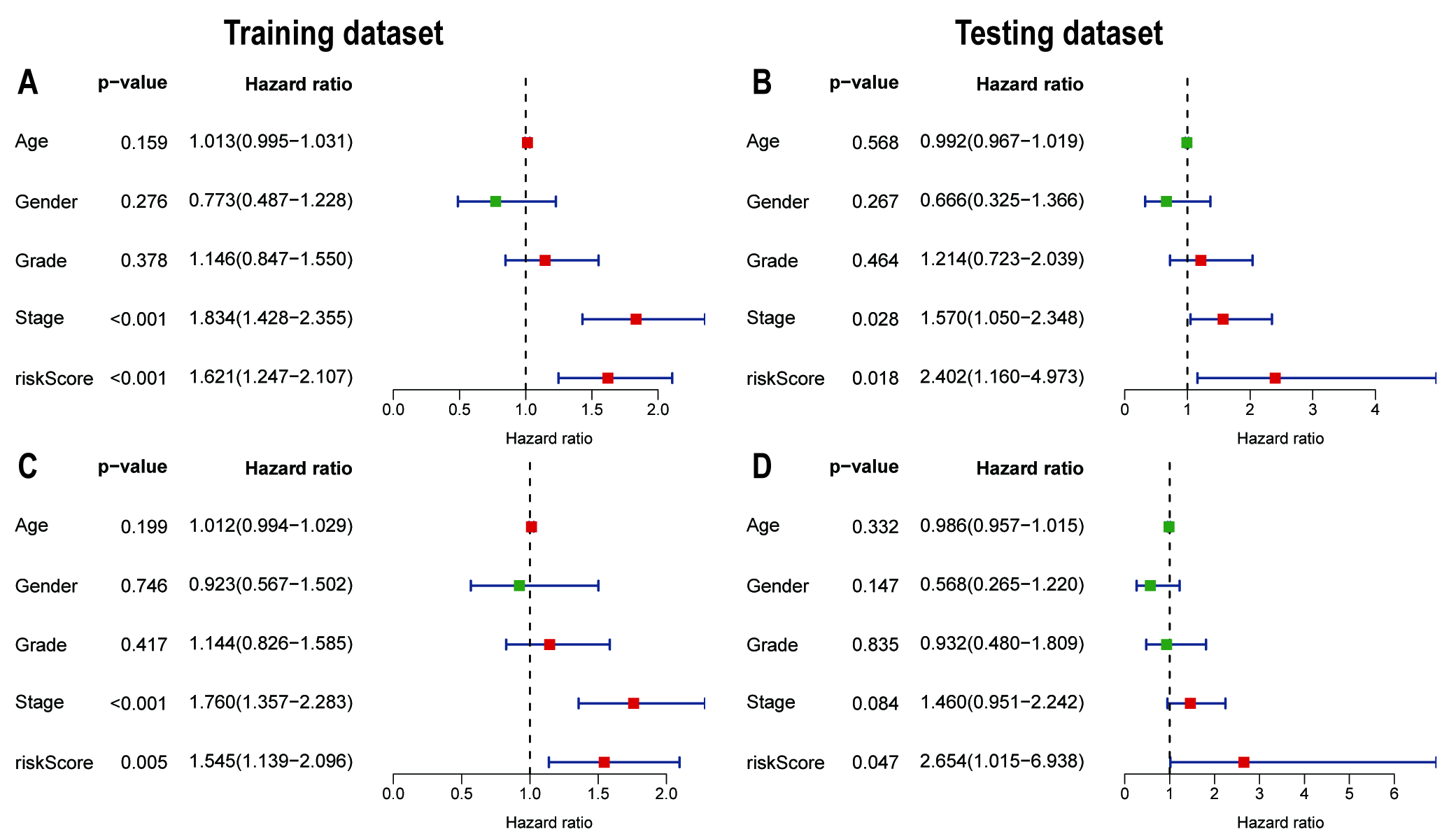

Supplement: Supplementary Figure 3 — Identify independent prognostic factors in the TCGA dataset. Forest plots indicate 95% confidence intervals (95% CI) for hazard ratios (HRs) and p-values for multivariate Cox regression in the (A) training and (B) testing datasets. [file Image_3.tif]

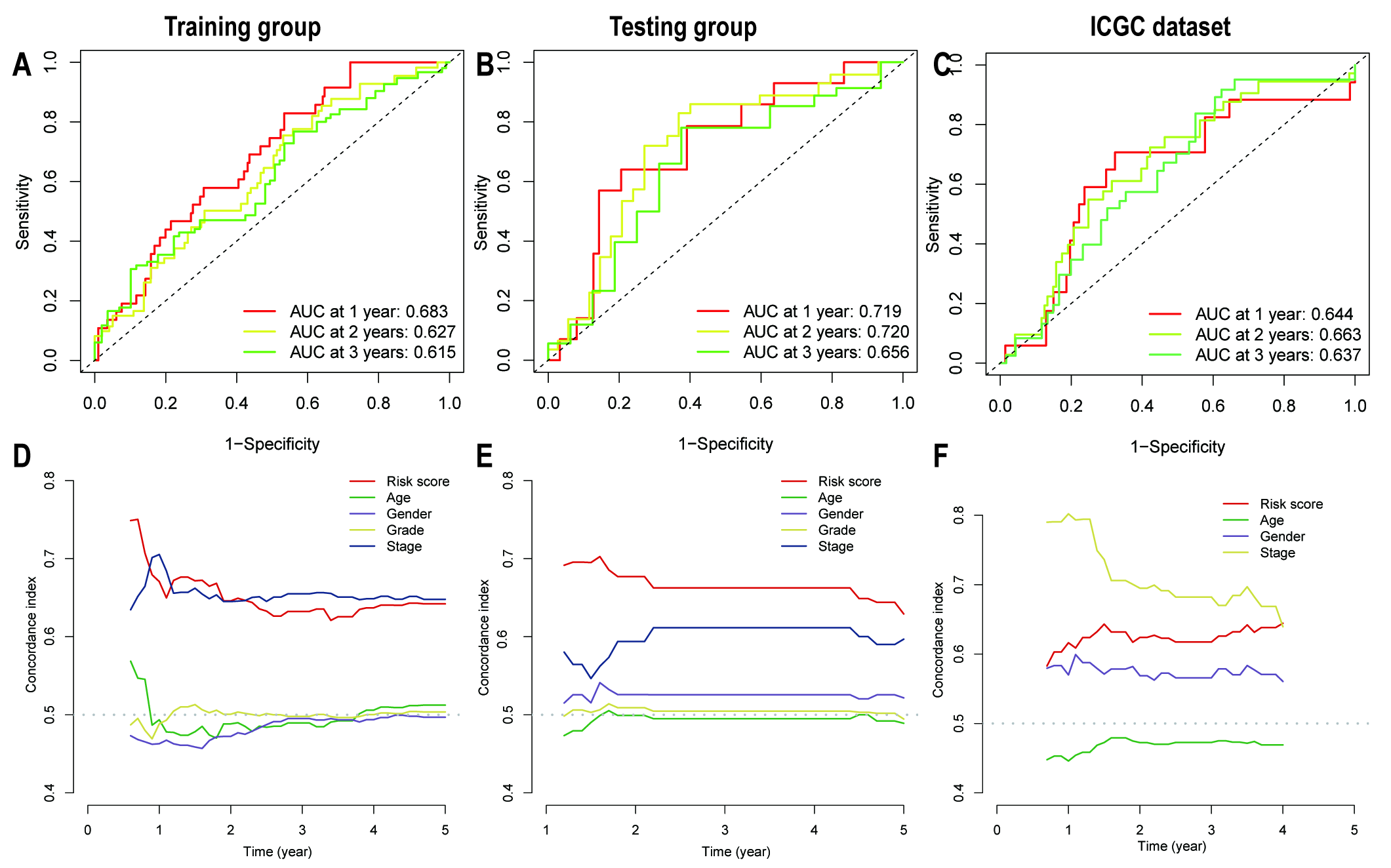

Supplement: Supplementary Figure 4 — Assessment of the accuracy of risk score and clinical characteristics in predicting the prognosis of patients with HCC. (A–C) ROC curve analysis for the training group, testing group, and external validation group respectively. (D, E) Concordance index analysis for the training group, testing group, and external validation group respectively. [file Image_4.tif]

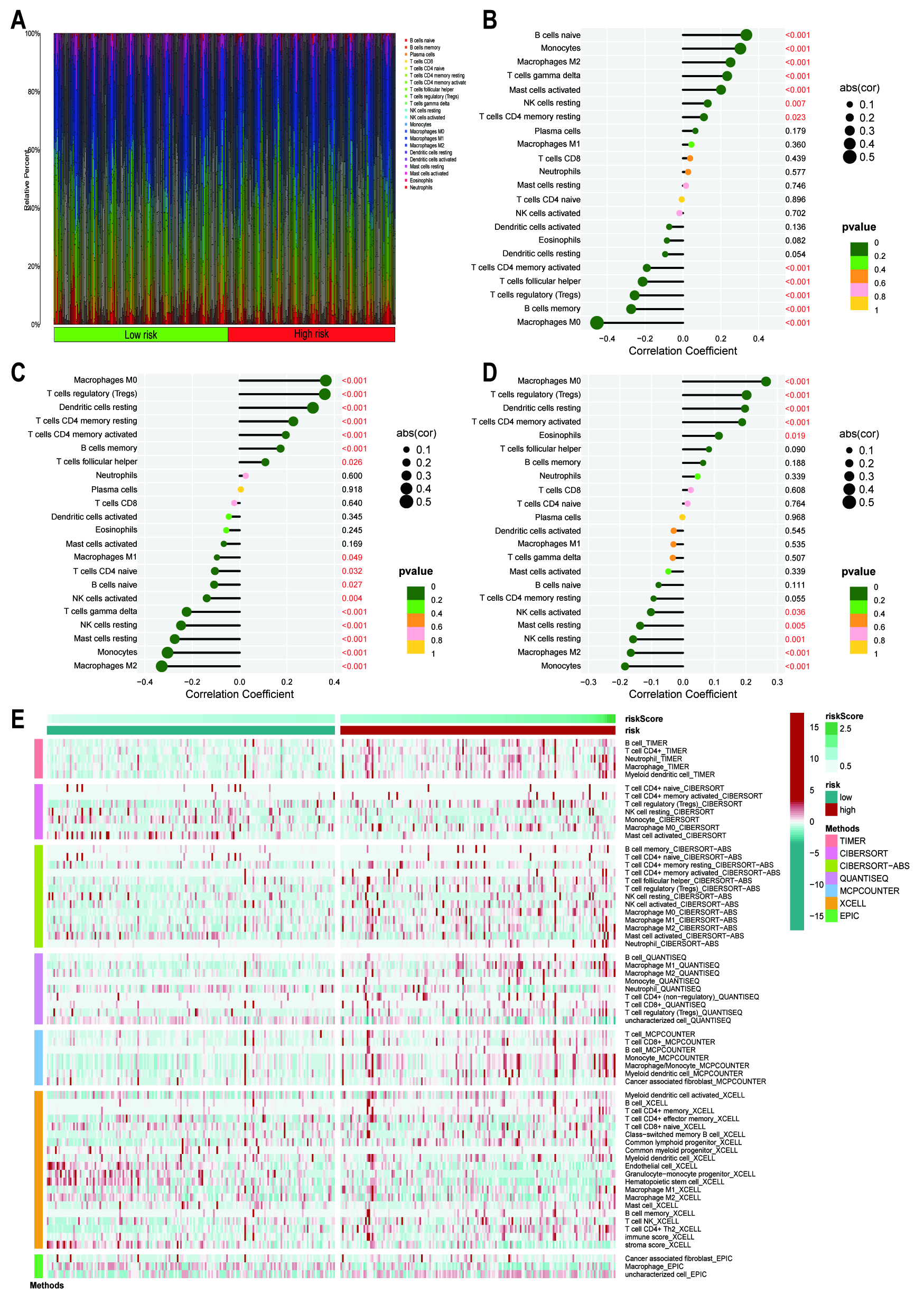

Supplement: Supplementary Figure 5 — Analysis of immune cell infiltration about risk score and prognostic genes in the TCGA database. (A) Overview of 22 immune cell infiltrations in the high- and low-risk groups. Correlation analysis of (B) CCL14, (C) CCL20, and (D) CCR3 with immune cells. (E) The landscape of immune cells infiltration between high- and low-risk groups by different algorithms. [file Image_5.tif]

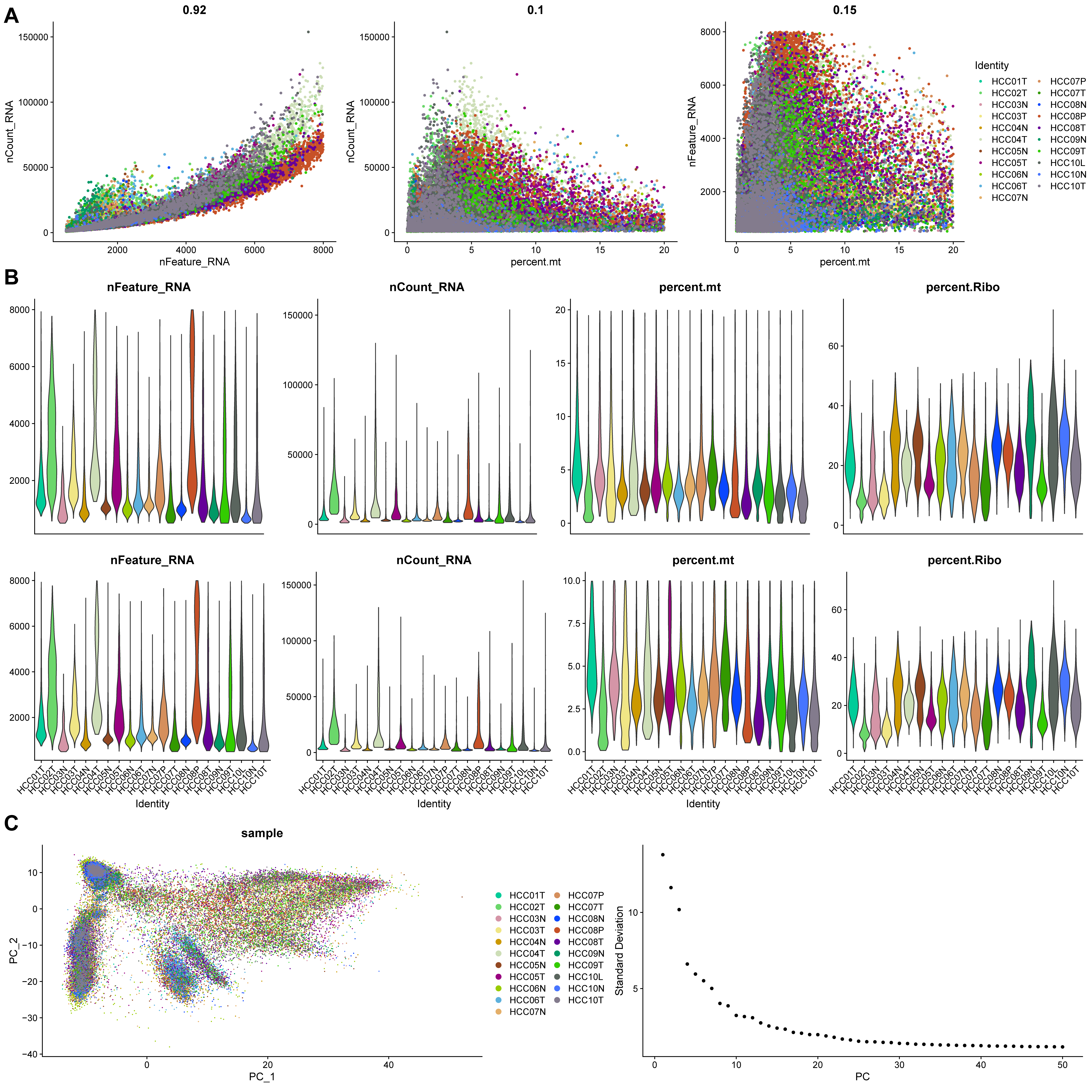

Supplement: Supplementary Figure 6 — The results of scRNA-seq data reprocessing. (A) Relationship between mitochondrial genes and UMI/mRNA amount, UMI and mRNA amount. (B-upper) Relationship between mRNA, UMI, mitochondrial content and rRNA content of each sample before filtering; (B-lower) Relationship between mRNA, UMI, mitochondrial content and rRNA content of each sample after filtering. (D) Principal component downscaled sample distribution and principal component anchor point plots. [file Image_6.tif]

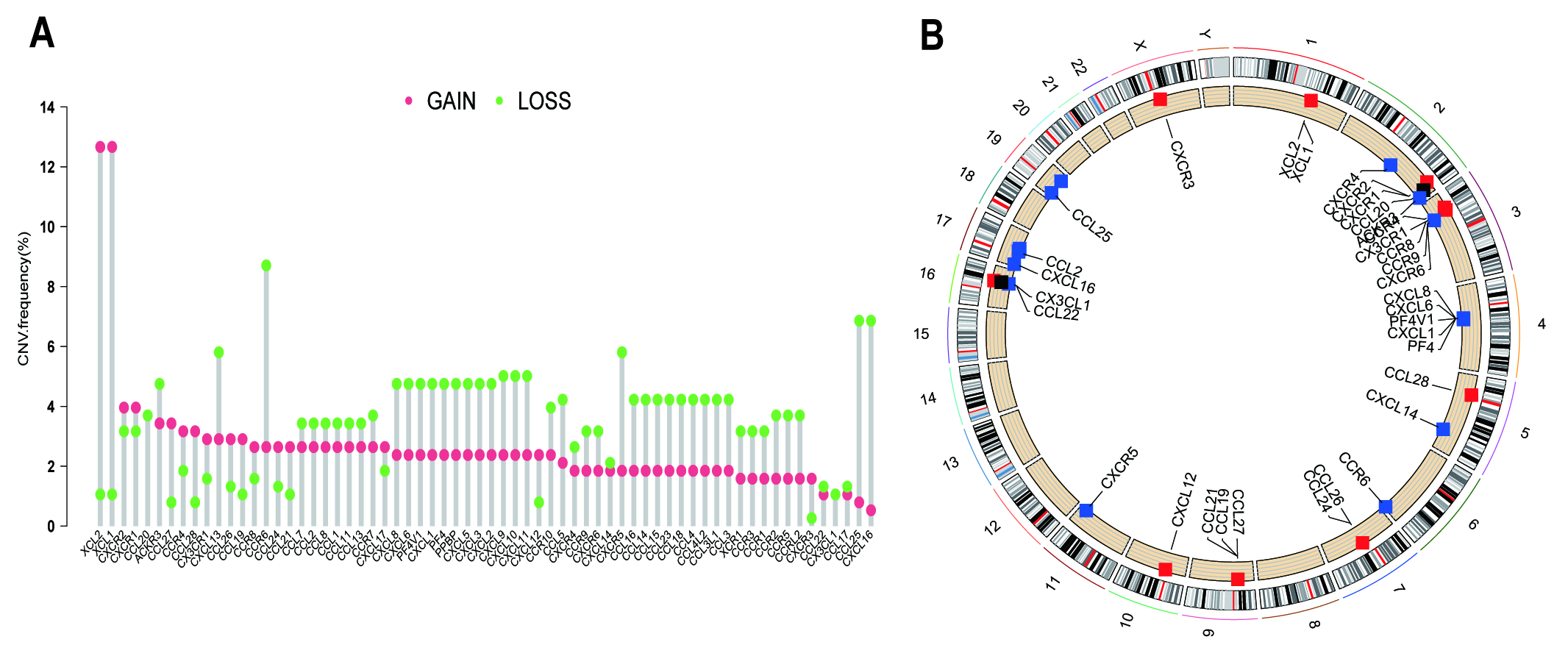

Supplement: Supplementary Figure 7 — The alterations of CRGs in the TCGA-LIHC cohort. (A) The proportions of copy number variations (CNVs) of CRGs in HCC. (B) Determining the chromosomal location of CRGs that occur CNVs in HCC. [file Image_7.tif]

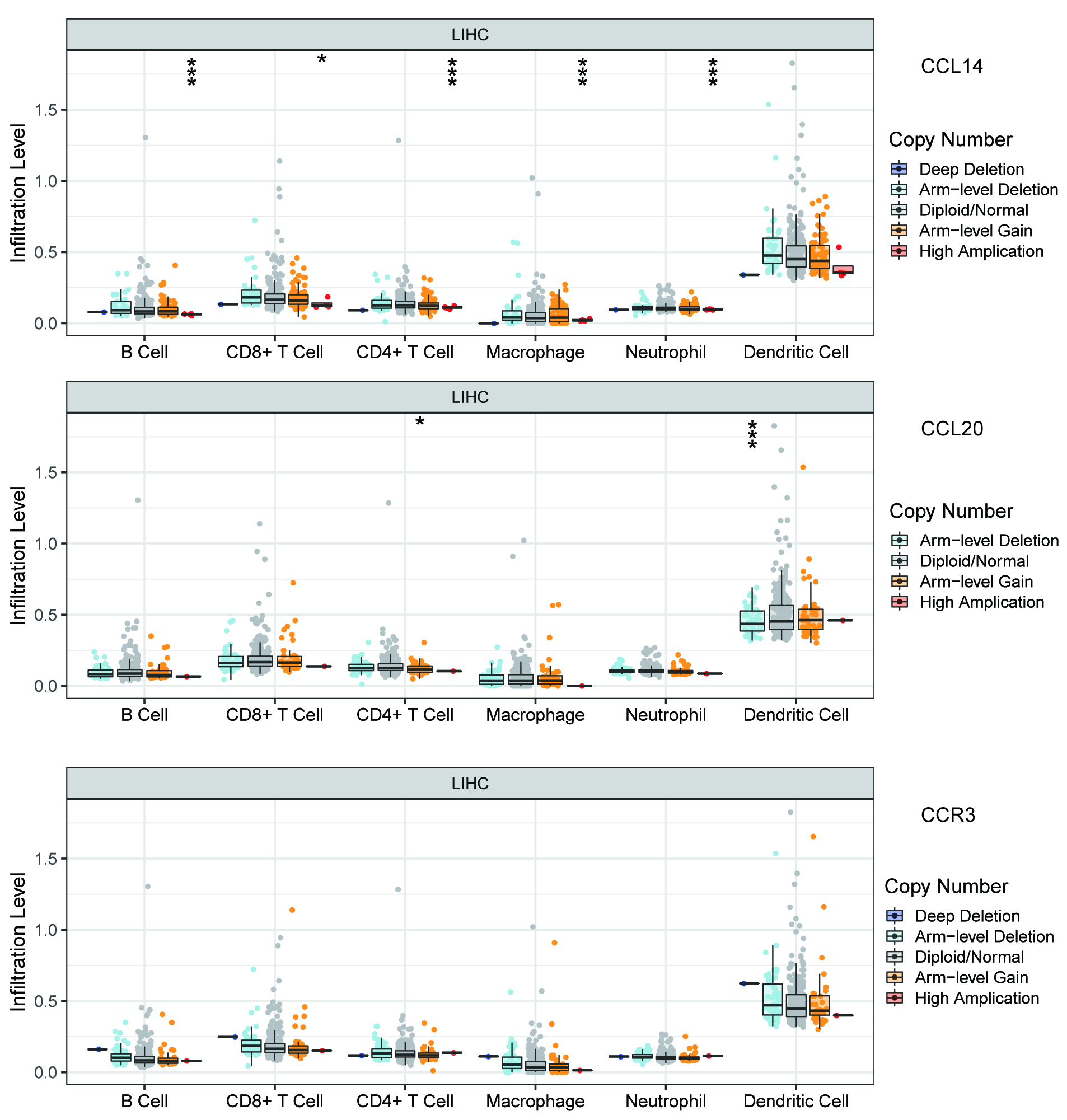

Supplement: Supplementary Figure 8 — Relationship between the CNVs types of prognostic genes and immune cell infiltration in the TCGA database. *P< 0.05, ***P< 0.001. [file Image_8.tif]
